# Huddling with families after disaster: Human resilience and social disparity

**DOI:** 10.1371/journal.pone.0273307

**Published:** 2022-09-28

**Authors:** Weiguang Wang, Natasha Z. Foutz, Guodong (Gordon) Gao

**Affiliations:** 1 Simon Business School, University of Rochester, Rochester, NY, United States of America; 2 McIntire School of Commerce, University of Virginia, Charlottesville, VA, United States of America; 3 R. H. Smith School of Business, University of Maryland, College Park, MD, United States of America; Neijiang Normal University, CHINA

## Abstract

Disasters, from hurricanes to pandemics, tremendously impact human lives and behaviors. Physical closeness to family post-disaster plays a critical role in mental healing and societal sustainability. Nonetheless, little is known about whether and how family colocation alters after a disaster, a topic of immense importance to a post-disaster society. We analyze 1 billion records of population-scale, granular, individual-level mobile location data to quantify family colocation, and examine the magnitude, dynamics, and socioeconomic heterogeneity of the shift in family colocation from the pre- to post-disaster period. Leveraging Hurricane Florence as a natural experiment, and Geographic Information System (GIS), machine learning, and statistical methods to investigate the shift across the landfall (treated) city of Wilmington, three partially treated cites on the hurricane’s path, and two control cities off the path, we uncover dramatic (18.9%), widespread (even among the partially treated cities), and enduring (over at least 3 months) escalations in family colocation. These findings reveal the powerful psychological and behavioral impacts of the disaster upon the broader populations, and simultaneously remarkable human resilience via behavioral adaptations during disastrous times. Importantly, the disaster created a gap across socioeconomic groups non-existent beforehand, with the disadvantaged displaying weaker lifts in family colocation. This sheds important lights on policy making and policy communication to promote sustainable family colocation, healthy coping strategies against traumatic experiences, social parity, and societal recovery.

## Introduction

Human history is constantly shaped and reshaped by natural disasters and human disasters, from volcanic eruptions and hurricanes, to wildfires [1] and pandemics [2, 3]. In the U.S. alone, 60,000 people lose lives in natural disasters each year [4, 5]. Family, the foundational unit of a society, plays a critical role in post-disaster physical, mental, economic recoveries, and broad societal sustainability. Specifically, family cohesion, the emotional bonding among family members [6], has been shown to promote physical and mental health [7], mitigate anxiety [8], affect discrimination [9], drinking and drug use [10]. One critical antecedent of family cohesion is family colocation, i.e., family members staying in close physical proximity. It offers a critical coping mechanism toward heightened mortality salience (i.e., heightened awareness of inevitable death) and perceived loss of control often experienced during disastrous times [11]. Nonetheless, little is known regarding how family colocation might change after a disaster, primarily due to a lack of large-scale tracking of human behavior. Compared to small-sample self-reports of family members being together, mobile location data embed continuous and natural observations of family colocations at a population-scale. These data hence offer unparalleled opportunities to examine temporal and spatial variations in family colocation, particularly around disastrous times when dynamics of family colocation and cohesion critically impact societal recovery.

Our study aims to quantify the magnitude and dynamics of the shift in family colocation after a disaster, and potential heterogeneity across socioeconomic groups, thus offering policy insights into disaster recovery, social disparity, and human well-being. Specifically, we are interested in: *(1) What are the magnitude and dynamics of the shift, if any, in family colocation after a disaster? (2) What are some of the mechanisms behind this shift? (3) Does a disaster alleviate or exacerbate social disparity, if any, in family colocation? (4) Which behavioral insights and policy implications can be drawn from these findings?*

To accomplish these, we examine a recent major disaster, Hurricane Florence, that made landfall on September 14, 2018 (treatment date) at Wilmington, NC (treated city), destroying 30,000 homes and causing 22 deaths and $24 billion damages [12–14]. Leveraging Geographic Information System (GIS) [15], machine learning, and Difference-in-Differences (DiD) statistical analyses on 1 billion location records, we compare the before-versus-after hurricane shift in family colocation at Wilmington, relative to two control cities off the hurricane’s path (and not impacted by the hurricane) and three partially treated cities on the path (and impacted mainly by low- to medium-level rainfalls). Family colocation is measured by each individual’s percentage of location records that are within a 50-meter vicinity of family members during each hour (details in the “Materials and Methods” section).

The analyses uncover a number of interesting findings. Compared to the control cities, Wilmington experienced a strong (18.9%) lift in family colocation after the hurricane, particularly through the mechanisms of weekday nighttime and in-home lifts in family colocation. The partially treated cities also displayed a strong lift, especially during the first month, revealing the powerful impact of the hurricane on the broader populations. Also, the lift in family colocation across all cities persisted, and even strengthened at Wilmington, over the next three months of our sampling period. Moreover, the disaster produced a previously non-existent gap in family colocation across socioeconomic groups, with the disadvantaged exhibiting a weaker lift. A series of robustness checks further validate these findings.

In summary, this research fills an important void of immense importance to a post-disaster society, by investigating the shift in family colocation as a coping mechanism toward traumatic experiences among the impacted populations. This research further enriches the psychological and behavioral theories on family togetherness and mortality salience by revealing a disaster as a crucial driver of the shift in family colocation, and increased family colocation as another novel response to heightened mortality salience. This study also takes an early step to enlist the newly available population-scale individual-level location data to examine the shift in post-disaster family relationship. The new GIS method proposed to identify family members and gauge family colocation is also applicable to a broad range of future geo-spatial and sociological research. Finally, our analyses quantify the magnitude, dynamics, and social disparity of a disaster’s impact on family colocation, hence shedding valuable lights on broad disaster management, policy-making and communication to advocate for family colocation, population well-being, and social equality.

## Materials and methods

### Literature

Our research primarily builds upon, and contributes to, two streams of literature, one on family cohesion and togetherness, and the other on mortality salience. We will review each below.

**Family cohesion and togetherness**. Family is the foundational unit of a society. Family cohesion, defined as the emotional bonding among family members [6], has been shown to impact many aspects of human behavior, such as discrimination [9], drinking, and drug use [10]. Elevated family cohesion may also mitigate anxiety [8], and support individuals’ physical health and mental health [7, 16]. A precursor of family cohesion is family togetherness, defined as family being together or spending time together, such as playing a video game together or going shopping together. Prior studies have also explored the effects of family togetherness on various outcomes, such as consumption [17]. Nonetheless, this literature has focused on the downstream impacts of family cohesion or togetherness. Our research, on the other hand, accentuates a potential antecedent or upstream driver of changes in family togetherness—the occurrence of a disaster.

The human history is laden with disasters, including natural disasters, such as meteorological (hurricanes), hydrological (floods), geo-physical (earthquakes), climatological (wildfires), and biological (COVID-19 pandemic) disasters; and human disasters, such as famines, social unrest, terrorist attacks, and wars. Disasters are commonly accompanied by deaths and other sufferings, rendering stronger needs for physical and mental supports, particularly from families [18]. Increased family colocation potentially fulfills such needs in the aftermath of a disaster, making our research on the potential shift in family colocation interesting and important.

**Mortality salience**. Disasters and accompanying death-related media also heighten mortality salience, that is, awareness of the inevitability of one’s death [19–21]. The psychological and behavioral literature on mortality salience has revealed that increased mortality salience may result in compulsive shopping or consumption, substance use, or other risky behaviors [22–24], particularly among the highly materialistic individuals [25] and those with low self-esteem [26]. A few studies also show that heightened mortality salience increases donations, and a sense of community or “we-ness” [27, 28]. This literature collectively suggests that these behavioral changes provide individuals with viable coping mechanisms to mitigate their escalated mortality salience.

Broadening these theories on mortality salience, our study uncovers that disaster-induced mortality salience also leads to another interesting behavioral change never documented previously—increased family colocation. Also importantly, unlike the literature that has largely focused on unhealthy coping mechanisms, escalated family colocation serves as a healthy coping mechanism against traumatic experiences. Furthermore, while the literature that has leveraged small-scale lab studies, our research offers a population-scale empirical evidence of individuals’ behavioral changes to mitigate the mental impact of a disaster. Particularly as the academic community has called for increased research amid the unprecedented COVID pandemic on individuals’ responses to disasters and threats [29], our findings offer important societal and policy implications and help mitigate disasters’ psychological impacts.

### Data

The treated city in our study is Wilmington, NC, where the landfall of Hurricane Florence took place. The partially treated and control cities are systematically selected from 1,923 cities in North Carolina and four adjacent states (GA, TN, SC, VA) with demographics most comparable to Wilmington: Savannah, GA; Newport News, VA; Charleston, SC; Chattanooga, TN; and Knoxville, TN. Specifically, Savannah and Newport News off the path of the hurricane serve as the control cities. The remaining three cities on the path of the hurricane are partially treated cities. Table 1 exhibits the summary statistics of the six cities under analyses.

**Table 1:** Demographics of the six cities under study.

	Wilmington	Charleston	Chattanooga	Knoxville	Savannah	Newport News
Population	119,045	134,875	179,139	187,347	146,444	179,388
Population <18 yrs	17.50%	17.40%	20.40%	18.90%	21.20%	23.30%
Population <65 yrs	15.50%	13.90%	15.60%	13.20%	12.80%	12.10%
Female	53.10%	52.10%	53.10%	52.10%	52.70%	51.50%
White	76.70%	74.40%	61.00%	75.20%	39.10%	49.00%
Black or African American	18.40%	21.90%	33.30%	17.50%	54.70%	40.70%
Hispanic or Latino	6.30%	2.90%	5.60%	5.60%	4.80%	8.60%
Median Household Income	$43,867	$61,367	$41,911	$36,331	$39,386	$51,082
Owner-occupied Housing	44.50%	54.40%	52.60%	45.30%	43.70%	50.00%
Location Records Per Person	761.45	901.18	721.61	614.53	719.42	725.65

Compared to the treated city of Wilmington, the control and partially treated cities experienced much lower impacts of the hurricane. For instance, among the 22 deaths caused by Florence, 15 occurred in NC, 4 in SC, and 3 in VA. Among the $24 billion estimated damages, NC accounted for $22 billion, followed by SC ($2 billion), VA ($200 million), and GA ($30 million) [30]. Aggregating the daily precipitation data (source: wunderground.com) between September 14 and 30 reveals that Wilmington endured the strongest impact (14.16 inches), followed by the partially treated cities, Chattanooga (5.9), Knoxville (5.85), and Charleston (1.51). The two control cities, Newport News and Savannah, had 0.75 and 0.32 inches, respectively. While the control and partially treated cities endured less infrastructure damages compared to Wilmington, they were potentially impacted psychologically, making it interesting to study if they also exhibited a similar shift in family colocation as Wilmington.

The mobile location data are curated in compliance with all privacy regulations, such as GDPR and CCPA, by a leading data aggregator from 400+ commonly used mobile apps across 32 categories in its app network. These apps have installed the aggregator’s proprietary software development kit (SDK) to help minimize smartphone battery drainage while tracking locations. Interested readers may refer to online materials (www.tamoco.com/blog/location-data-info-faq-guide) for more information about the common industrial practice of mobile location tracking. These 32 categories of apps cover a wide range of an individual’s lifestyle, such as entertainment, finance, food and drink, health and fitness, map and navigation, shopping, and weather. There is no change in the list of mobile apps in the aggregator’s app network during our sampling period. The specific names of these apps are not provided to preserve consumer privacy and business confidentiality.

These apps track opt-in individuals’ locations every five to fifteen minutes, or when there is a significant change in a person’s geo-coordinates. The data cover a quarter of the U.S. population and are representative of the U.S. population based on the data aggregator’s detailed analyses. Each data record comprises of an anonymized ID of the individual, timestamp, longitude, latitude, speed, and dwell time at a visited location. Each individual’s home is determined by the most frequent location 2–5AM Tuesdays to Fridays (removing Mondays due to potential spillover activities from weekends). Given the tracking accuracy of the data (20–50 meters), each individual’s family members include those with home locations within the same cluster of a 50-meter radius based on hierarchical clustering [31, 32]. Family colocation during each hour is then measured by each individual’s percentage of location records within a 50-meter vicinity of any family member during that hour. To mitigate potential measurement errors, we subsequently test a number of alternative measures of family colocation; and the findings remain robust (cf. S1 File).

### Methodology

We employ the Difference-in-differences (DiD) statistical analysis to quantify the shift in family colocation before versus after the hurricane at Wilmington relative to the control (and subsequently partially treated) cities. DiD is a popular approach for causal inference with strong methodological foundations [33–36]. Specifically, the DiD method is to leverage a treated group exposed to the treatment and a control group not influenced by the treatment. By comparing the difference between the treated and control groups before versus after the treatment, the DiD model could identify the effect of the treatment. In this study, Wilmington is the treated group (treated by the hurricane’s landfall), whereas Savannah and Newport News constitute the control group. As depicted in S3 Fig, we aim to quantify the impact of the hurricane on family colocation by comparing the change in family colocation before versus after the hurricane’s landfall of the treated group versus that of the control group. The corresponding regression model of our DiD approach is described by Eq 1:

$$\begin{array}{c} Y_{ij}=\alpha_{i}+\beta_{1}{Post}_{j}+\beta_{2}{Treat}_{i}\times {Post}_{j}+\beta_{3}X_{j}+\epsilon_{ij}. \end{array}$$ (1)

Here *Y*_*ij*_ is the percentage of an individual *i*’s location records during hour *j* when *i* colocates with his/her family member(s). The range of *Y*_*ij*_ is [0, 1]. *α*_*i*_ is the individual fixed effect. *Post*_*j*_ is a dummy equal to 1 for the post-hurricane period and 0 otherwise. *Treat*_*i*_ is a dummy indicating whether *i* is from the treated city of Wilmington. The interaction term *Treat*_*i*_ × *Post*_*j*_ is of key interest and captures the treatment effect of the hurricane on the shift in family colocation at Wilmington relative to the control cities. *X*_*j*_ includes a vector of control variables, such as the hour-of-the-day. We also use the robust and clustered standard errors in the regression analyses to more rigorously address heteroskedasticity and potential violation of the independence assumption of the error term. We subsequently further control for the day- or city-fixed effects as robustness checks; and all findings sustain. When comparing Wilmington with both the control and partially treated cities, we further include *PartTreat*_*i*_ × *Post*_*j*_ in the above equation. All models are estimated using ordinary least square (OLS); and the results are further verified with alternative specifications, such as Fractional Logistic Regression (cf. S1 File).

Next, we leverage the Difference-in-Difference-in-Differences (DDD) analysis to examine the heterogeneous treatment effect of the hurricane on family colocation across socioeconomic groups:

$$\begin{array}{c} Y_{ij}=\beta_{1}{Treat}_{i}+\beta_{2}{Post}_{j}+\beta_{3}Z_{i}+\beta_{4}{Treat}_{i}\times {Post}_{j}+\beta_{5}{Treat}_{i}\times Z_{i}+\beta_{6}{Post}_{j}\times Z_{i}+\beta_{7}{{Treat}_{i}\times Post}_{j}\times Z_{i}+\beta_{8}X_{j}+\epsilon_{ij}, \end{array}$$ (2)

where *Y*_*ij*_, *Post*_*j*_, *Treat*_*i*_, and *X*_*j*_ remain identical to those in Eq 1; *Z*_*i*_ is a socioeconomic factor of *i*’s home census block group (CBG), as the location data are not associated with the individual-level socioeconomic factors to preserve privacy. The three-way interaction *Treat*_*i*_ × *Post*_*j*_ × *Z*_*i*_ captures the heterogeneous treatment effect, i.e., the moderating effect of the socioeconomic factor on the main treatment effect of the hurricane on family colocation.

## Results

### Model-free evidence

Hurricane Florence is one of the latest major disasters hitting the U.S. Its impact was not nationwide or global; nor did it entail extended voluntary or mandatory stay-at-home, or unemployment that would inherently escalate family colocation. Hence it provides a relatively clean context to study potential shifts in family colocation after a disaster with minimal confounding factors, and allows us to leverage its differential impacts upon different cities. The sample period spans 75 days (6/30–9/12) before the landfall (9/14) and 77 days (9/15–11/30) after. We do not analyze 9/13 and 9/14 when the residents were preparing for the landfall. We also exclude the individuals with less than 10 records in the pre- or post-treatment period to ensure that sufficient data are present to observe any behavioral changes.

**Fig 1:**
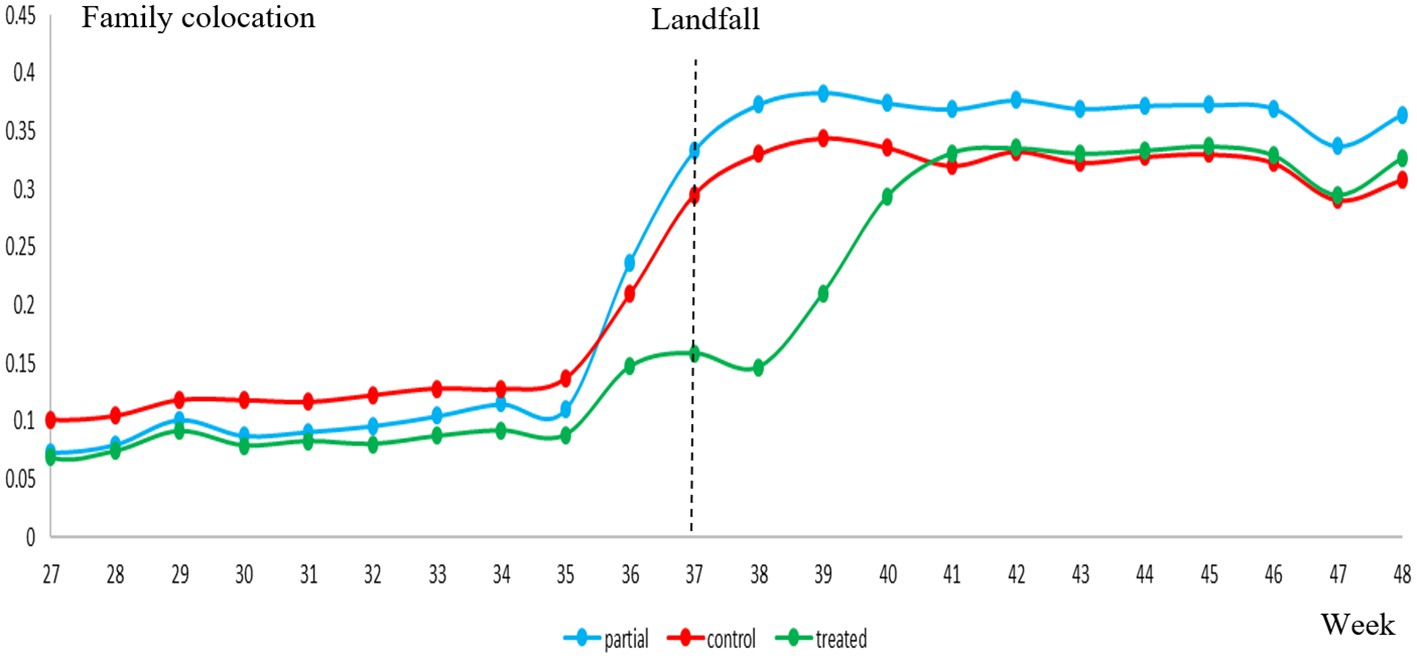
Family colocation over time.

**Fig 2:**
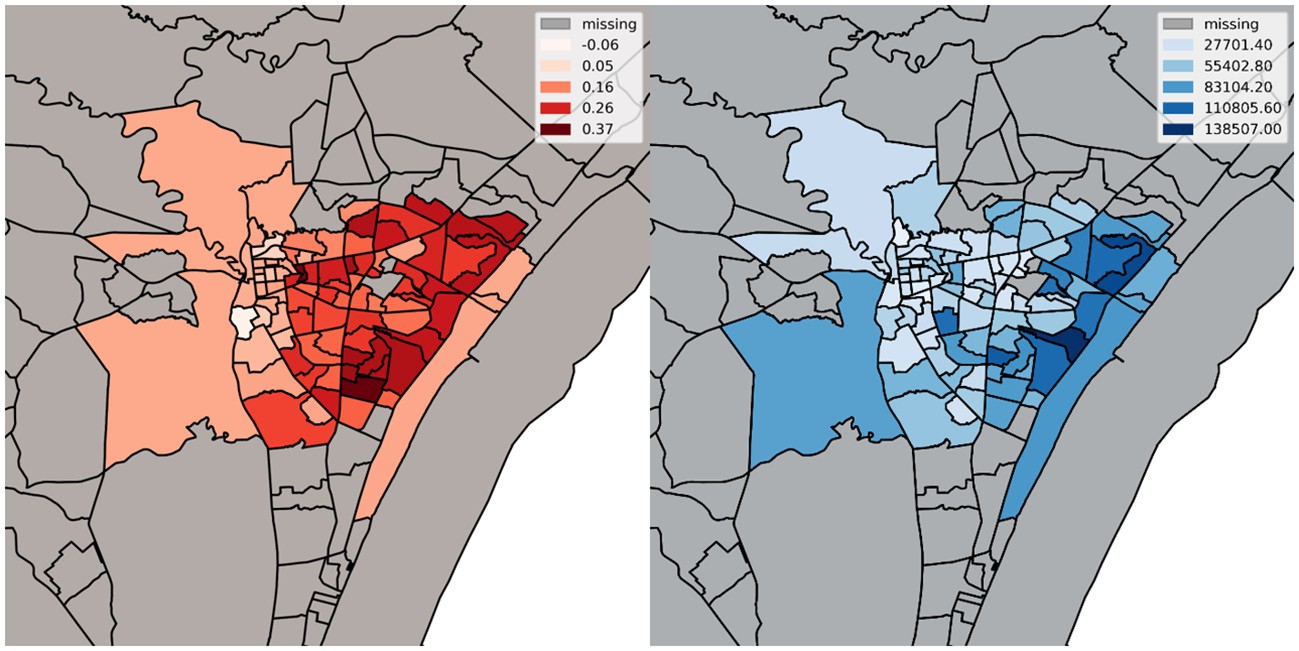
Shift in Family Colocation across CBGs (Left) of Different Median Household Income (Right) at Wilmington. This figure is generated using open source Python and its matplotlib library, in addition to the Open Census Data dataset from SafeGraph, which is available under the CC0 license.

Fig 1 shows that all cities experienced lifts in family colocation after the hurricane, indicating the strong psychological and behavioral impact of the hurricane on the broader populations. These lifts persisted over at least 3 months of our sampling period, with an initial dip for Wilmington, potentially driven by the residents’ immediate recovery activities. Moreover, Fig 2 reveals that higher-income census block groups (CBGs hereafter) at Wilmington experienced higher lifts, offering initial evidence of the heterogeneous impacts of the hurricane across socioeconomic groups.

### Magnitude of the shift in family colocation

Compared to the control cities, Wilmington experienced a strong 18.9% lift in family colocation from its pre-hurricane level of 10.36% (.0196/.1036 = 18.9%, Table 2). This pronounced lift is potentially driven by the elevated mortality salience and perceived loss of control after the hurricane, hence escalated desire for family comfort, affection, and social acceptance [19, 20]. Interestingly, when comparing the treated, partially treated, and control cities altogether, we find that the partially treated cities displayed an even stronger lift in family colocation (.0234) than treated city (.0193; Wald Test: *p*<0.01), revealing the powerful impact of the disaster upon the populations beyond the landfall city. Our subsequent mechanism analyses reveal that this finding is primarily driven by the stronger lift in family colocation over the weekend nighttime at the partially treated cities relative to Wilmington during the first month after the hurricane (cf. *Exploring potential mechanisms* below).

**Table 2:** Magnitude of the shift in family colocation.

	Treated vs Control	Treated vs Partially Treated and Control
Post	.0785***	.0788***
	(.0016)	(.0016)
Treat × Post	.0196***	.0193***
	(.0039)	(.0039)
PartTreat × Post		.0234***
		(.0021)
Individual Fixed Effect	Yes	Yes
Controls	Yes	Yes
# Obs.	36,020,422	93,631,371
# Users	49,322	123,298

Robust and clustered standard errors are in parentheses.

*** *p* <0.01,

** *p* <0.05,

* *p* <0.1.

### Dynamics of the shift in family colocation

Table 3 exhibits the treatment effects over the first week and first, second, third month after the hurricane, respectively. The sample ends three months after the landfall to circumvent the impact of winter holidays on family colocation. Consistent with Fig 1, family colocation at Wilmington decreased significantly relative to the control cities during the first week after the hurricane (-.1200, *p*<0.01), as family members likely scattered to perform recovery activities, such as some shopping for groceries while others acquiring home repair materials. Nonetheless, over the subsequent three months, Wilmington displayed stronger and persistent lifts in family colocation, albeit at a decreasing rate, than the control cities, revealing a long-lasting impact of the disaster on family colocation. Our further investigation also shows that these findings cannot be explained by infrastructure restoration or unemployment after the hurricane, as revealed by the location data and additional government statistics (cf. *Supplemental Information—Infrastructure restoration and unemployment after Hurricane Florence*).

**Table 3:** Dynamics of the shift in family colocation.

	Treated vs Control			
	1st week	1st month	2nd month	3rd month
Post	.0669***	.0834***	.0729***	.0542***
	(.0014)	(.0015)	(.0017)	(.0018)
Treat×Post	-.1200***	.0023***	.0397***	.0450***
	(.0043)	(.0035)	(.0043)	(.0044)
PartTreat×Post				
Individual FE	Yes	Yes	Yes	Yes
Controls	Yes	Yes	Yes	Yes
# Obs.	11,362,782	19,506,976	20,030,180	14,903,946
# Users	48,415	48,954	48,869	48,770
	Treated vs Partially Treated and Control			
	1st week	1st month	2nd month	3rd month
Post	.0672***	.0836***	.0729***	.0542***
	(.0014)	(.0015)	(.0017)	(.0018)
Treat×Post	-.1200***	-.0022	.0395***	.0450***
	(.0043)	(.0035)	(.0043)	(.0044)
PartTreat×Post	.0057***	.0180***	.0200***	.0197***
	(.0017)	(.0019)	(.0022)	(.0022)
Individual FE	Yes	Yes	Yes	Yes
Controls	Yes	Yes	Yes	Yes
# Obs.	26,755,175	49,772,315	49,387,158	35,956,406
# Users	121,291	122,448	122,248	122,006

Robust and clustered standard errors are in parentheses.

*** *p* <0.01,

** *p* <0.05,

* *p* <0.1.

### Exploring potential mechanisms

To explore some of the potential mechanisms underlying these findings, we further examine the timing of family colocation: time-of-the-day and day-of-the-week. That is, we seek to understand whether the post-hurricane escalation in family colocation at Wilmington is driven by the increase in family colocation during the daytime or nighttime, weekday or weekend.

This exploration is motivated by the literature on circadian rhythm. Specifically, individuals may desire and attain varied levels of family colocation at different times-of-the-day and days-of-the-week because of circadian rhythm and societal rhythm. Prior studies on circadian rhythm show that humans perform at the lowest level at night [37], thus experiencing more negative emotions and potentially desiring more family colocation at night. The social norm of working during daytime and weekdays also makes family colocation during nighttime and weekends more feasible. We hence group time-of-the-day into daytime (sunrise-sunset) and nighttime (sunset-sunrise), determined for each city and each day based on the data from timeanddate.com. We then examine whether the lift in family colocation at Wilmington arises from that over weekday daytime, weekday nighttime, weekend daytime, or weekend nighttime.

When compared to the control cities, Wilmington experienced the lifts in family colocation both during the daytime and nighttime (cf. significantly positive three-way interactions in Column 1 of S1 Table). Both Wilmington and the partially treated cities exhibit stronger lifts of family colocation during the nighttime than daytime (Column 2 of S1 Table; Wald Test *p*<0.1). And family colocation at the partially treated cities increased significantly more during the weekends than weekdays, whereas the effect is not as significant for Wilmington.

We hence further examine the dynamics pertaining to the above findings (S2 Table). In the initial week after the hurricane, family colocation at Wilmington fell below the control and partially treated cities. Nonetheless, in the second and third months, Wilmington displayed stronger lifts than the control and partially treated cities across all times-of-the-day and days-of-the-week (Wald test *p*<0.01). Recall that Wilmington experienced greater infrastructure damages than the other cities, and hence greater needs for recovery. As a result, in the first week after the landfall, as families engaged in recovery activities, for instance, some family members purchasing home repair materials while others removing neighborhood debris, family cocolation at Wilmington actually fell below the other cities. After the recovery completed, Wilmington surpassed both the control and partially treated cities in family colocation at all times-of-the-day and days-of-the-week, indicating a stronger longer-term impact of the hurricane on the landfall city than on other cities.

### Socioeconomic divergence in the shift in family colocation

Family colocation could be heterogeneous across socioeconomic groups before or after a disaster. For instance, lower-income individuals could have more irregular or extended work hours, thus colocating less. In contrast, those of higher socioeconomic status enjoy more disposable income and more flexible work schedule [38, 39], and hence can afford more colocations with families if they chose so after the hurricane hit. To illustrate such potential socioeconomic divergence, we use the CBG-level median household income as an example. Specifically, we compare those with higher-income (living in a Wilmington CBG with a median income higher than the city median of $52,204) and lower income (below median). The significant three-way interaction (Column 1 of Table 4) suggests that compared with the control cities, Wilmington CBGs with lower income experienced weaker lifts in family colocation after the hurricane (.0430). This finding is particularly striking since the disadvantaged populations displayed similar levels of family colocation before the disaster hit (S2 Fig). In other words, the disaster essentially generated a new dimension of social disparity in terms of family colocation.

**Table 4:** Socioeconomic divergence in the shift in family colocation.

	All	Weekend	Weekday	Day	Night	AtHome	OutHome
Treat × Post × Income	.0430**	.0221	.0482***	.0388***	.0496**	-.00169	.0462***
	(.0174)	(.0224)	(.0168)	(.0136)	(.0226)	(.0344)	(.00955)
Income	.00156	-.0525***	.0158**	-.0100*	.0134	-.0483***	.0388***
	(.00756)	(.00964)	(.00728)	(.00553)	(.0105)	(.0158)	(.00240)
Post×Income	.1020***	.158***	.0875***	.0509***	.140***	.113***	.0596***
	(.0083)	(.0110)	(.00797)	(.00694)	(.0108)	(.0155)	(.00467)
Treat×Income	-.0016	.0215	-.00764	.0126	-.0166	-.0579*	.00251
	(.0139)	(.0173)	(.0136)	(.0104)	(.0193)	(.0334)	(.00516)
Treat	-.0537***	-.0705***	-.0490***	-.0391***	-.0726***	-.0391*	-.0103***
	(.0090)	(.0114)	(.00863)	(.00660)	(.0126)	(.0221)	(.00248)
Post	.1060***	.101***	.107***	.0840***	.132***	.203***	.0386***
	(.0055)	(.00707)	(.00527)	(.00464)	(.00706)	(.0100)	(.00286)
Treat×Post	-.0027	.00822	-.00550	-.00674	.00813	.0558**	-.0297***
	(.0115)	(.0146)	(.0111)	(.00893)	(.0150)	(.0232)	(.00498)
Controls	Yes	Yes	Yes	Yes	Yes	Yes	Yes
# Obs.	35,069,915	7,916,394	27,153,521	16,254,535	18,815,380	12,864,363	17,833,383
# Users	47,711	42,710	47,228	47,484	45,691	29,360	47,645

Robust and clustered standard errors are in parentheses.

*** *p* <0.01,

** *p* <0.05,

* *p* <0.1.

We further conduct a number of analyses to gain greater insights into the potential reasons of such social disparity (Column 2–7 of Table 4). First, the social disparity is stronger over weekdays than weekends (Columns 2 and 3), as higher-income individuals benefit from reduced income pressure and more flexible work schedules, and can thus afford increased colocations with families during weekdays. On weekends, this difference between the higher- versus lower-income individuals dissipates. Second, the social disparity is slightly stronger over the nighttime than daytime (Columns 4 and 5), as the higher-income individuals have more flexible work schedules and may join families earlier or spend more time with families in the evenings. Third, the social disparity occurs primarily out-of-home (Columns 6 and 7), suggesting that higher-income individuals co-locate with families more when going out. Overall, one may portray a higher-income individual who returns home earlier or goes out more with families (instead of for instance colleagues or friends) after work on weekdays since experiencing the hurricane landfall.

Finally, we conduct further analyses using an alternative measure of family colocation, as we have done in S7 and S8 Tables, S1 Fig: each individual’s average distance from home during a given hour. We find that those of higher socioeconomic status stay closer to home after the hurricane, hence having greater opportunities to be with families. Specifically, this distance reduces by 831 meters (from 2,025 to 1,194) for the higher-income, whereas only 306 meters (from 2,110 to 1,804) for the lower-income.

### Robustness tests

To further validate the findings, we conduct a series of robustness tests, such as exploring residential migrations across cities, alternative model specifications, and alternative measures of family colocation (cf. S1 File). All key findings remain.

## Conclusion

### Contributions and policy implications

This research examines a topic of essential importance to society: a disaster’s impact and human resilience. We empirically demonstrate, for the first time at the individual level and for a massive population, surprisingly dramatic, widespread, and enduring escalation of family colocation post-disaster. These findings furnish initial yet persuasive evidence of the powerful psychological and behavioral impact of a disaster, and concurrently remarkable human resilience via behavioral adaptation, hence of immense academic and societal importance. The multitude of cutting-edge spatial-temporal analytic methods has also unleashed the power of terabyte-sized, naturally and continuously observed (instead of self-reported and static) human mobility.

Also importantly, while past studies have documented various vulnerabilities of the disadvantaged during disastrous times [40], this research reveals a new dimension of vulnerability. A weaker lift in family colocation could put the disadvantaged at higher risks, or prolong their recovery from a disaster. The study hence sheds additional lights on the latest debates about social disparity during the COVID-19 pandemic. Moreover, our findings illuminate the need for policy makers to close the gap in family colocation across socioeconomic groups during disastrous times, offering vulnerable populations greater opportunities and affordability to colocate and bond with families, such as more flexible work hours. These policies could engender profound impacts across numerous vital societal domains, such as climate change, crime, divorce, education, juvenile behavior, and so on [41].

Our research further enriches the literature on family togetherness and family cohesion by discovering disasters as a driving force of the shift in family colocation. This study also broadens the theories on mortality salience and human responses to traumatic experiences by revealing that escalated family colocation offers a novel, and more importantly, healthy coping mechanism. Our findings on human resilience further enrich the on-going discussions on resilience across various domains in our society during the global pandemic.

Lastly, this research takes an early step to unleash the power of the newly available population-scale and individual-level location big data in studying topics of societal importance. Our proposed method to identify family members and gauge family colocation is also applicable to a broad spectrum of future geo-spatial and sociological research.

### Limitations and future research

Despite the contributions, our research presents a number of limitations, and hence invites future explorations. For instance, as the location data cover one quarter of the U.S. population, not every single family member may be included, making our measure of the family colocation a lower-bound estimate. Meanwhile, this results in an even stronger evidence of the profound and persistent impact of the hurricane on family colocation. Also, our study measures offline-only family colocation, and hence captures a lower bound of the post-disaster elevation in family togetherness, which could also take place online, such as via phone calls or social media interactions. Future research may integrate online and offline data, ideally enriched with detailed family interactions, to more comprehensively gauge family colocation and cohesion. In addition, our data end at three months after the disaster. It will be interesting to quantify an even longer-term impact of a disaster on family colocation. Lastly, it is a worthwhile future effort to dive more deeply into the potential mechanisms and mitigation strategies of the discovered social disparities.

## Supporting information

S1 File (PDF)

S1 Table Exploring mechanism 1: Heterogeneous treatment effects across time-of-the-day and day-of-the-week. (PDF)

S2 Table Exploring mechanism 1: Dynamics of heterogeneous treatment effects across time-of-the-day and day-of-the-week. (PDF)

S3 Table Migrations across cities. (PDF)

S4 Table Alternative specification: Fractional logistic regression. (PDF)

S5 Table Magnitude of the shift in family colocation: DV = Dummy. (PDF)

S6 Table Dynamics in the shift in family colocation: DV = Dummy. (PDF)

S7 Table Magnitude of the shift in family colocation (Measured by distance from home). (PDF)

S8 Table Dynamics of the shift in family colocation (Measured by distance from home). (PDF)

S1 Fig Family colocation (Measured by distance from home) over time. (TIF)

S2 Fig Family colocation over time by income. (TIF)

S3 Fig Difference-in-Differences model. (TIF)

S4 Fig Unemployment rate of Wilmington, NC. (TIF)
