# Supplementary material for: Huddling with families after disaster: Human resilience and social disparity
Source: PLoS One. 2022 Sep 28;17(9):e0273307. doi: 10.1371/journal.pone.0273307 (PMC9518864; doi:10.1371/journal.pone.0273307)
Supplement: S1 File — (PDF) [file pone.0273307.s001.pdf]

Exploring potential mechanisms

S1 Table-S2 Table display the results related to the potential mechanisms underlying the main findings: time-of-the-day and day-of-the-week.

Robustness test 1: migrations

To investigate whether the findings on family colocation are partly driven by the hurricane related residential migrations, we calculate the number (percentage) of the individuals from each city who appeared in the other five cities in our sample after the hurricane (S3 Table). The small percentages of the migrants suggests that the discovered effects are unlikely driven by, for instance, the Wilmington residents more likely to colocate with families self-selected to stay at Wilmington, or the residents from other cities migrated into Wilmington after the hurricane.

Robustness test 2: alternative specification

In addition to the ordinary least square (OLS) estimation of the proposed model, we also estimate a fractional logistic regression when comparing Wilmington and the control cities, given that the dependent variable is a percentage measure. S4 Table shows consistent findings as those in the main manuscript.

Robustness test 3: alternative measures of family colocation

Alternative measure 1: family colocation as a dummy variable. To address a potential concern that different individuals might have different number of location records per hour, we also examine a binary dependent variable, equal to 1 if an individual is within

a 50-meter vicinity of any family member at least once in a given hour; and 0 otherwise. We then re-run all analyses using logistic regression and all findings sustain (S5 Table-S6 Table).

Alternative measure 2: family colocation with home. We further calculate an alternative measure of family colocation, as colocation with home, i.e., an individual's average distance in meters from his/her home in a given hour ("distance from home" hereafter). It gauges an individual's attachment to home and further alleviates a potential concern over missing family members not covered by the location data. S1 Fig shows that distance from home reduced more for Wilmington after the hurricane than for the other cities. Also consistent with the main finding (Table 1), the significantly negative coefficient of $Treat \times Post$ (-95.11, p<0.01, S7 Table) suggests a great reduction in the distance from home at Wilmington relative to the control cities after the hurricane. All other findings related to the dynamics of the effects (S8 Table) also remain consistent.

Socioeconomic divergence in the shift in family colocation

S2 Fig visualizes the family colocation for each socioeconomic group over time, before and after the hurricane, for Wilmington and the control cities. Consistently, the family colocation is comparable for the high- and low-income groups before the hurricane. However, a gap emerged after the hurricane: the low-income groups experienced weaker lifts in family colocation, particularly at Wilmington.

Infrastructure restoration and unemployment after Hurricane Florence

We also conduct a thorough search on the infrastructure restoration. First, the disruption caused by Hurricane Florence occurred primarily over the first few days after the landfall. The emergency status of North Carolina was largely removed after two weeks as daily life returned to normal. Second, while the National Weather Services reported that the widespread flash flooding had inundated neighborhoods and closed roads [1], the flood waters receded 10 days after the landfall and the North Carolina Office of the Governor reopened the highways on September 23 [2, 3]. Third, the National Weather Services documented that the power outage at Wilmington was

restored within two weeks. Fourth, the Hurricane Florence After Action Report released by the New Hanover County, NC also stated that the emergency operations, including the pre-landfall preparation, remained in effect for only 21 days. Overall, the infrastructure restoration was completed rather quickly after the landfall.

Moreover, according to the U.S. Bureau of Labor Statistics [4], the unemployment rate at Wilmington, NC did not fluctuate out of ordinary during September and the few months thereafter S4 Fig. Also, Wilmington residents reportedly returned to work within a week after the hurricane.

Hence, our findings are unlikely driven by the infrastructure restoration. Nonetheless, the psychological impact, which is the focus of our research, can be longer-lasting. For instance, the HOPE4NC Crisis Counseling Program has served 221,079 hurricane survivors since the landfall, among whom 56,134 received support even after July 1, 2019, nearly one year after the landfall [5]. Therefore, our finding on the elevated family colocation after the hurricane captures a key longer-term psychological impact of the disaster.

References

1. NWS-Wilmington. Hurricane Florence: September 14, 2018; 2018. Available from: `https://www.weather.gov/ilm/HurricaneFlorence`.
2. GovernorNC. I-95 through North Carolina now open, Gov. Cooper announces; 2018. Available from: `https://governor.nc.gov/news/i-95-through-north-carolina-now-open-gov-cooper-announces`.
3. GovernorNC. As floodwaters recede, state focuses on recovery; 2018. Available from: `https://governor.nc.gov/news/floodwaters-recede-state-focuses-recovery`.
4. USBLS. Economy at a glance, Wilmington, NC. U.S. Bureau of Labor Statistics; 2022. Available from: `https://www.bls.gov/eag/eag.nc_wilmington_msa.htm`.
5. HOPE4NC. Hope 4 NC program aids hurricane recovery in NC; 2019. Available from: `https://rhahealthservices.org/hope-4-nc-program-aids-hurricane-recovery-in-nc/`.
