# Supplementary material for: Huddling with families after disaster: Human resilience and social disparity
Source: PLoS One. 2022 Sep 28;17(9):e0273307. doi: 10.1371/journal.pone.0273307 (PMC9518864; doi:10.1371/journal.pone.0273307)
Supplement: S1 Table — (PDF) [file pone.0273307.s002.pdf]

S1 Table. Exploring Mechanism 1: Heterogeneous Treatment Effects across Time-of-the-day and Day-of-the-week

	Treated vs. Control	Treated vs. Part vs. Control
weekendDay	0.0669***	0.0682***
	(0.00161)	(0.00156)
weekdayDay	0.0455***	0.0468***
	(0.00164)	(0.00178)
weekendNight	-0.0479***	-0.0479***
	(0.00123)	(0.00123)
Treat × Post × weekendDay	0.0130***	0.0130***
	(0.00461)	(0.00461)
Treat × Post × weekdayDay	0.0212***	0.0212***
	(0.00407)	(0.00407)
Treat × Post × weekendNight	0.0236***	0.0236***
	(0.00561)	(0.00561)
Treat × Post × weekdayNight	0.0298***	0.0298***
	(0.00475)	(0.00475)
PartTreat × Post × weekendDay		0.0262***
		(0.00257)
PartTreat × Post × weekdayDay		0.0196***
		(0.00209)
PartTreat × Post × weekendNight		0.0371***
		(0.00324)
PartTreat × Post × weekdayNight		0.0264***
		(0.00254)
Post × weekendDay	0.0682***	0.0688***
	(0.00197)	(0.00197)
Post × weekdayDay	0.0147***	0.0153***
	(0.00161)	(0.00162)
Post × weekendNight	0.136***	0.136***
	(0.00257)	(0.00254)
Post × weekdayNight	0.128***	0.127***
	(0.00205)	(0.00203)
Treat × weekendDay	0.00336	-0.0165
	(0.0386)	(0.0354)
Treat × weekdayDay	0.0185	-0.00138
	(0.0386)	(0.0353)
Treat × weekendNight	-0.0291	-0.0490
	(0.0387)	(0.0355)
Treat × weekdayNight	-0.0353	-0.0552
	(0.0386)	(0.0354)
PartTreat × weekendDay		-0.0435**
		(0.0190)
PartTreat × weekdayDay		-0.0265
		(0.0190)
PartTreat × weekendNight		-0.0566***
		(0.0191)
PartTreat × weekdayNight		-0.0380**
		(0.0191)
Individual Fixed Effect	Yes	Yes
# Obs.	36,020,422	93,631,371
# Users	49,322	123,298

Robust and clustered standard errors are in parentheses. *** $p < 0.01$, ** $p < 0.05$, * $p < 0.1$.
