# Supplementary material for: Huddling with families after disaster: Human resilience and social disparity
Source: PLoS One. 2022 Sep 28;17(9):e0273307. doi: 10.1371/journal.pone.0273307 (PMC9518864; doi:10.1371/journal.pone.0273307)
Supplement: S2 Table — (PDF) [file pone.0273307.s003.pdf]

S2 Table. Exploring Mechanism 1: Dynamics and Duration of Heterogeneous Treatment Effects across Time-of-the-day and Day-of-the-week

	Treated vs Control				Treated vs Partially Treated and Control			
	1st week	1st month	2nd month	3rd month	1st week	1st month	2nd month	3rd month
weekendDay	-0.00206	0.0343***	0.0482***	0.0361***	-0.00294**	0.0275***	0.0469***	0.0354***
	(0.00156)	(0.00152)	(0.00154)	(0.00161)	(0.00149)	(0.00149)	(0.00153)	(0.00154)
weekdayDay	-0.0279***	0.0118***	0.0246***	0.0108***	-0.0289***	0.00491***	0.0233***	0.0100***
	(0.00132)	(0.00144)	(0.00153)	(0.00159)	(0.00163)	(0.00166)	(0.00174)	(0.00174)
weekendNight	-0.0431***	-0.0469***	-0.0457***	-0.0436***	-0.0431***	-0.0469***	-0.0457***	-0.0436***
	(0.00117)	(0.00120)	(0.00121)	(0.00119)	(0.00117)	(0.00121)	(0.00121)	(0.00119)
Treat × Post × weekendDay	-0.0678***	0.000246	0.0317***	0.0386***	-0.0675***	0.000529	0.0317***	0.0383***
	(0.00643)	(0.00430)	(0.00503)	(0.00554)	(0.00643)	(0.00430)	(0.00503)	(0.00554)
Treat × Post × weekdayDay	-0.0536***	0.0121***	0.0405***	0.0439***	-0.0536***	0.0123***	0.0405***	0.0434***
	(0.00440)	(0.00365)	(0.00449)	(0.00452)	(0.00440)	(0.00365)	(0.00449)	(0.00452)
Treat × Post × weekendNight	-0.122***	0.00637	0.0421***	0.0537***	-0.123***	0.00605	0.0422***	0.0542***
	(0.00836)	(0.00537)	(0.00605)	(0.00659)	(0.00837)	(0.00537)	(0.00605)	(0.00659)
Treat × Post × weekdayNight	-0.193***	0.000249	0.0535***	0.0593***	-0.194***	-0.000357	0.0534***	0.0598***
	(0.00563)	(0.00449)	(0.00520)	(0.00546)	(0.00563)	(0.00449)	(0.00520)	(0.00545)
PartTreat × Post × weekendDay					-0.00728**	0.0211***	0.0223***	0.0171***
					(0.00301)	(0.00241)	(0.00276)	(0.00307)
PartTreat × Post × weekdayDay					-0.000764	0.0158***	0.0161***	0.0121***
					(0.00191)	(0.00195)	(0.00225)	(0.00230)
PartTreat × Post × weekendNight					0.00909**	0.0322***	0.0313***	0.0348***
					(0.00383)	(0.00309)	(0.00344)	(0.00372)
PartTreat × Post × weekdayNight					0.00675***	0.0199***	0.0241***	0.0257***
					(0.00245)	(0.00241)	(0.00273)	(0.00284)
Post × weekendDay	0.0168***	0.0656***	0.0549***	0.0490***	0.0170***	0.0652***	0.0550***	0.0491***
	(0.00231)	(0.00185)	(0.00216)	(0.00243)	(0.00231)	(0.00186)	(0.00216)	(0.00243)
Post × weekdayDay	0.0155***	0.0193***	0.000371	-0.0114***	0.0153***	0.0191***	0.000539	-0.0111***
	(0.00152)	(0.00152)	(0.00177)	(0.00183)	(0.00152)	(0.00152)	(0.00177)	(0.00183)
Post × weekendNight	0.0661***	0.138***	0.130***	0.105***	0.0667***	0.138***	0.129***	0.104***
	(0.00297)	(0.00244)	(0.00277)	(0.00301)	(0.00297)	(0.00243)	(0.00274)	(0.00298)
Post × weekdayNight	0.126***	0.134***	0.124***	0.0958***	0.126***	0.134***	0.124***	0.0954***
	(0.00197)	(0.00194)	(0.00224)	(0.00234)	(0.00198)	(0.00193)	(0.00221)	(0.00231)
Treat × weekendDay	-0.0126	0.00898	-0.0328	-0.0380	-0.0140	1.64e-05	-0.0550*	-0.0349
	(0.0368)	(0.0366)	(0.0412)	(0.0262)	(0.0295)	(0.0320)	(0.0330)	(0.0213)
Treat × weekdayDay	0.00474	0.0247	-0.0161	-0.0207	0.00345	0.0159	-0.0383	-0.0175
	(0.0368)	(0.0366)	(0.0412)	(0.0262)	(0.0295)	(0.0320)	(0.0330)	(0.0214)
Treat × weekendNight	-0.0443	-0.0230	-0.0652	-0.0698***	-0.0458	-0.0320	-0.0874***	-0.0668***
	(0.0369)	(0.0367)	(0.0413)	(0.0263)	(0.0297)	(0.0321)	(0.0330)	(0.0214)
Treat × weekdayNight	-0.0493	-0.0289	-0.0699*	-0.0738***	-0.0508*	-0.0378	-0.0921***	-0.0708***
	(0.0369)	(0.0367)	(0.0412)	(0.0263)	(0.0297)	(0.0321)	(0.0330)	(0.0214)
PartTreat × weekendDay					-0.0368*	-0.0423**	-0.0384*	-0.0344*
					(0.0196)	(0.0182)	(0.0197)	(0.0203)
PartTreat × weekdayDay					-0.0249	-0.0268	-0.0227	-0.0199
					(0.0196)	(0.0182)	(0.0197)	(0.0203)
PartTreat × weekendNight					-0.0496**	-0.0553***	-0.0514***	-0.0477**
					(0.0197)	(0.0183)	(0.0199)	(0.0204)
PartTreat × weekdayNight					-0.0366*	-0.0382**	-0.0343*	-0.0320
					(0.0197)	(0.0183)	(0.0198)	(0.0204)
Individual Fixed Effect	Yes	Yes	Yes	Yes	Yes	Yes	Yes	Yes
Controls	Yes	Yes	Yes	Yes	Yes	Yes	Yes	Yes
# Obs.	11,362,782	19,506,976	20,030,180	14,903,946	26,755,175	26,755,175	49,772,315	49,772,315
# Users	48,415	48,954	48,869	48,770	121,291	121,291	122,448	122,448

Robust and clustered standard errors are in parentheses. *** $p < 0.01$, ** $p < 0.05$, * $p < 0.1$.
