# Supplementary material for: Huddling with families after disaster: Human resilience and social disparity
Source: PLoS One. 2022 Sep 28;17(9):e0273307. doi: 10.1371/journal.pone.0273307 (PMC9518864; doi:10.1371/journal.pone.0273307)
Supplement: S3 Table — (PDF) [file pone.0273307.s004.pdf]

S4 Table. Migrations across Cities

City	# (%) Appearing in Other Cities	# (%) Appearing in Wilmington
Wilmington (treated)	165 (2.16%)	NA
Newport News (control)	99 (5.14%)	14 (.07%)
Savannah (control)	1347 (5.97%)	85 (.38%)
Charleston (partially treated)	1012 (4.05%)	51 (.20%)
Chattanooga (partially treated)	2168 (7.89%)	12 (.04%)
Knoxville (partially treated)	1991 (8.02%)	13 (.05%)
