# Supplementary material for: Huddling with families after disaster: Human resilience and social disparity
Source: PLoS One. 2022 Sep 28;17(9):e0273307. doi: 10.1371/journal.pone.0273307 (PMC9518864; doi:10.1371/journal.pone.0273307)
Supplement: S4 Table — (PDF) [file pone.0273307.s005.pdf]

S5 Table. Alternative Specification: Fractional Logistic Regression

	Overall	1st week	1st month	2nd month	3rd month
Post	1.0040***	.9910***	1.0360***	1.0180***	.9020***
	(.0011)	(.0018)	(.0012)	(.0012)	(.0014)
Treat	-.4830***	-.4820***	-.4830***	-.4830***	-.4820***
	(.0028)	(.0028)	(.0028)	(.0028)	(.0028)
Treat × Post	.4230***	-1.056***	.2100***	.5060***	.5320***
	(.0030)	(.0101)	(.0035)	(.0033)	(.0036)
# Obs.	36,020,422	11,362,782	19,506,976	20,030,180	14,903,946

Robust and clustered standard errors are in parentheses. *** $p < 0.01$, ** $p < 0.05$, * $p < 0.1$.
