# Supplementary material for: Huddling with families after disaster: Human resilience and social disparity
Source: PLoS One. 2022 Sep 28;17(9):e0273307. doi: 10.1371/journal.pone.0273307 (PMC9518864; doi:10.1371/journal.pone.0273307)
Supplement: S5 Table — (PDF) [file pone.0273307.s006.pdf]

S6 Table. Magnitude of the Shift in Family Colocation: DV = Dummy

	Treated vs Control	Treated vs Partially Treated and Control
Post	1.009***	1.006***
	(0.0137)	(0.0137)
Treat	-0.533***	-0.532***
	(0.0387)	(0.0386)
PartTreat		0.0111
		(0.0177)
Treat × Post	0.446***	0.446***
	(0.0381)	(0.0380)
PartTreat × Post		0.204***
		(0.0167)
# Obs.	36,020,422	93,631,371
# Users	49,322	123,298

Robust and clustered standard errors are in parentheses. *** p <0.01, ** p <0.05, * p <0.1.
