# Supplementary material for: Huddling with families after disaster: Human resilience and social disparity
Source: PLoS One. 2022 Sep 28;17(9):e0273307. doi: 10.1371/journal.pone.0273307 (PMC9518864; doi:10.1371/journal.pone.0273307)
Supplement: S6 Table — (PDF) [file pone.0273307.s007.pdf]

S7 Table. Dynamics and Duration in the Shift in Family Colocation: DV = Dummy

	Treated vs Control			
	1st week	1st month	2nd month	3rd month
Post	0.998***	1.049***	1.021***	0.900***
	(0.0132)	(0.0134)	(0.0142)	(0.0147)
Treat	-0.533***	-0.533***	-0.533***	-0.532***
	(0.0386)	(0.0386)	(0.0387)	(0.0386)
Treat × Post	-1.090***	0.218***	0.539***	0.564***
	(0.0519)	(0.0369)	(0.0392)	(0.0400)
# Obs.	11,362,782	19,506,976	20,030,180	14,903,946
# Users	48,415	48,954	48,869	48,770

	Treated vs Partially Treated and Control			
	1st week	1st month	2nd month	3rd month
Post	0.994***	1.046***	1.046***	0.897***
	(0.0132)	(0.0133)	(0.0133)	(0.0147)
Treat	-0.531***	-0.532***	-0.532***	-0.531***
	(0.0386)	(0.0386)	(0.0386)	(0.0385)
PartTreat	0.0110	0.0111	0.0111	0.0109
	(0.0176)	(0.0176)	(0.0176)	(0.0176)
Treat × Post	-1.087***	0.217***	0.217***	0.563***
	(0.0518)	(0.0368)	(0.0368)	(0.0399)
PartTreat × Post	0.192***	0.184***	0.184***	0.242***
	(0.0163)	(0.0164)	(0.0164)	(0.0179)
# Obs.	26,755,175	49,772,315	49,387,158	35,956,406
# Users	121,291	122,448	122,248	122,006

Robust and clustered standard errors are in parentheses. *** p <0.01, ** p <0.05, * p <0.1.
