# Supplementary material for: Huddling with families after disaster: Human resilience and social disparity
Source: PLoS One. 2022 Sep 28;17(9):e0273307. doi: 10.1371/journal.pone.0273307 (PMC9518864; doi:10.1371/journal.pone.0273307)
Supplement: S7 Table — (PDF) [file pone.0273307.s008.pdf]

S8 Table. Magnitude of the Shift in Family Colocation (Measured by Distance from Home)

	Treated vs Control	Treated vs Partially Treated and Control
Post	0.687	-5.885
	(11.92)	(12.32)
Treat × Post	-95.11***	-105.3***
	(22.57)	(23.00)
PartTreat × Post		-5.778
		(16.07)
# Obs.	36,020,422	93,631,371
# Users	49,322	123,298

Robust and clustered standard errors are in parentheses. *** $p < 0.01$, ** $p < 0.05$, * $p < 0.1$.
