# Supplementary material for: Huddling with families after disaster: Human resilience and social disparity
Source: PLoS One. 2022 Sep 28;17(9):e0273307. doi: 10.1371/journal.pone.0273307 (PMC9518864; doi:10.1371/journal.pone.0273307)
Supplement: S8 Table — (PDF) [file pone.0273307.s009.pdf]

**S9 Table. Dynamics and Duration of the Shift in Family Colocation (Measured by Distance from Home)**

| | Treated vs Control | | | |
|---|---|---|---|---|
| | 1st week | 1st month | 2nd month | 3rd month |
| Post | -69.42*** | -19.03* | -20.38 | 2.353 |
| | (12.17) | (11.12) | (13.72) | (16.61) |
| Treat × Post | 172.2*** | -67.79*** | -90.43*** | -100.2*** |
| | (45.09) | (21.99) | (24.39) | (27.41) |
| # Obs. | 11,362,782 | 19,506,976 | 20,030,180 | 14,903,946 |
| # Users | 48,415 | 48,954 | 48,869 | 48,770 |

| | Treated vs Partially Treated and Control | | | |
|---|---|---|---|---|
| | 1st week | 1st month | 2nd month | 3rd month |
| Post | -71.16*** | -22.90** | -23.71* | -1.383 |
| | (12.30) | (11.27) | (13.89) | (16.72) |
| Treat × Post | 172.3*** | -76.25*** | -95.26*** | -105.2*** |
| | (45.13) | (22.24) | (24.56) | (27.57) |
| PartTreat × Post | 65.30*** | -2.405 | 2.595 | -16.36 |
| | (15.54) | (14.47) | (17.60) | (20.61) |
| # Obs. | 26,755,175 | 49,772,315 | 49,387,158 | 35,956,406 |
| # Users | 121,291 | 122,448 | 122,248 | 122,006 |

Robust and clustered standard errors are in parentheses. *** $p < 0.01$, ** $p < 0.05$, * $p < 0.1$.
