# Supplementary figures and images for: Huddling with families after disaster: Human resilience and social disparity
Source: PLoS One. 2022 Sep 28;17(9):e0273307. doi: 10.1371/journal.pone.0273307 (PMC9518864; doi:10.1371/journal.pone.0273307)

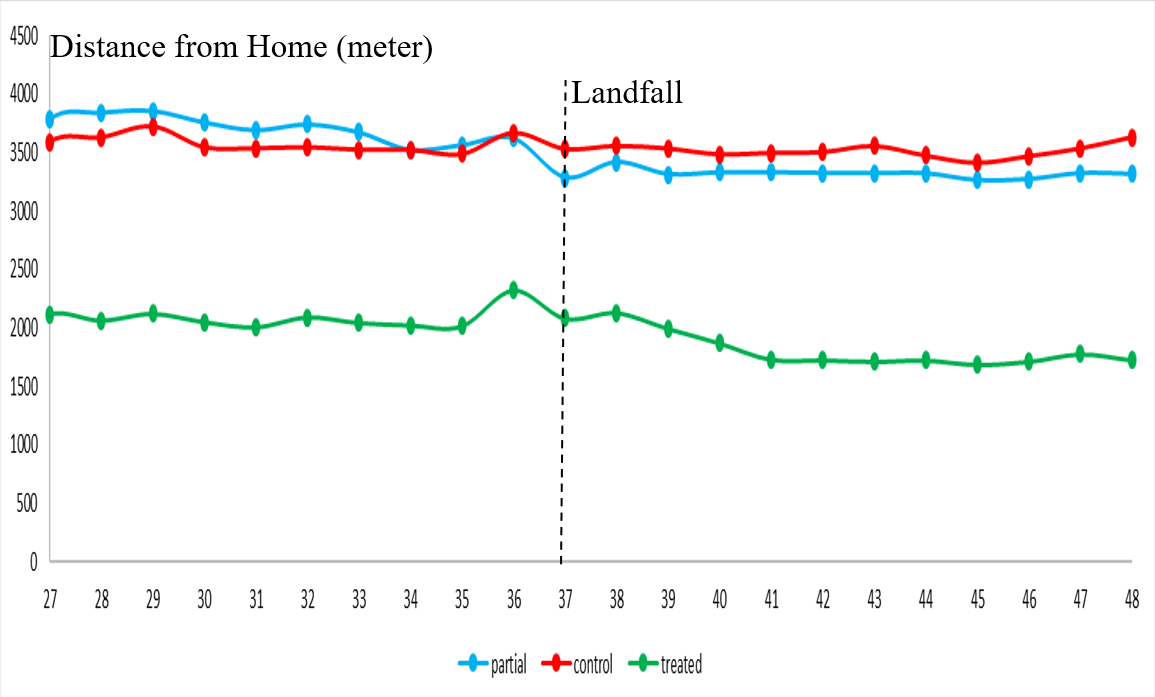

Supplement: S1 Fig — (TIF) [file pone.0273307.s010.tif]

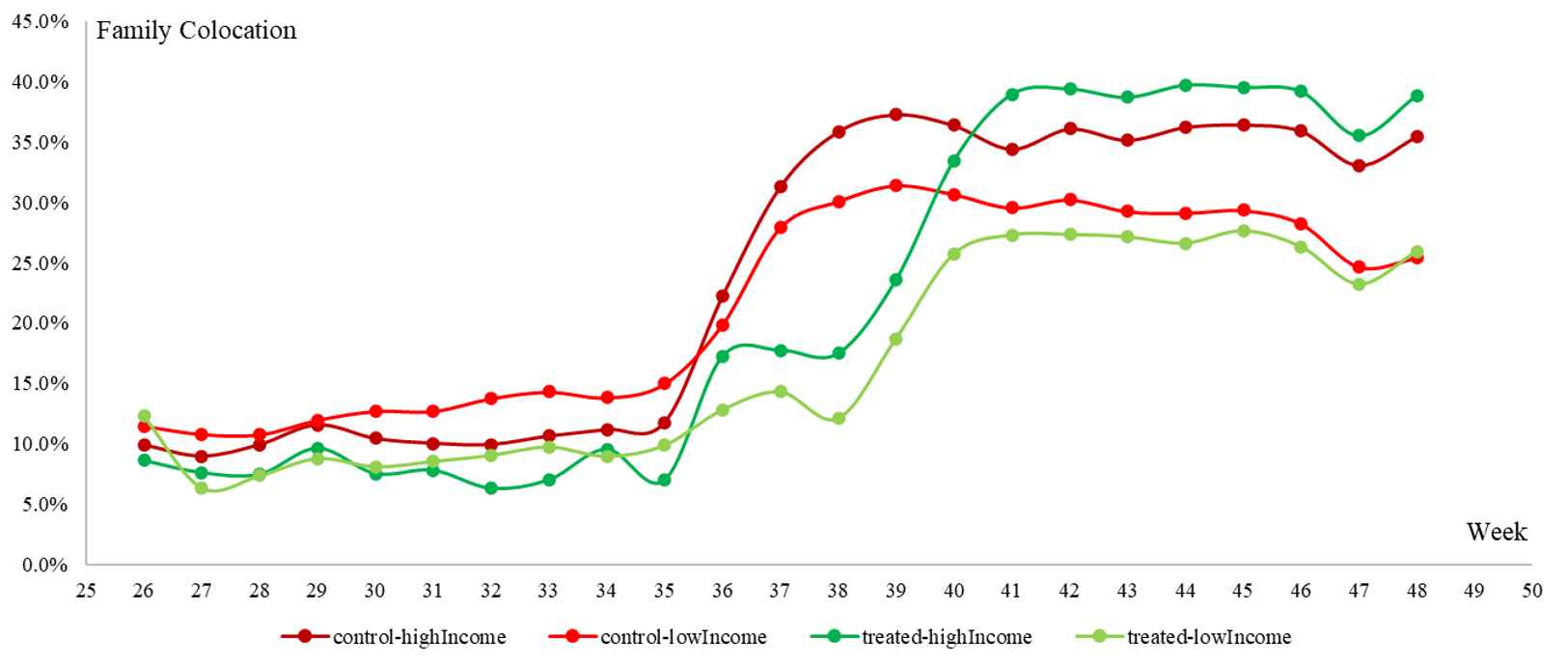

Supplement: S2 Fig — (TIF) [file pone.0273307.s011.tif]

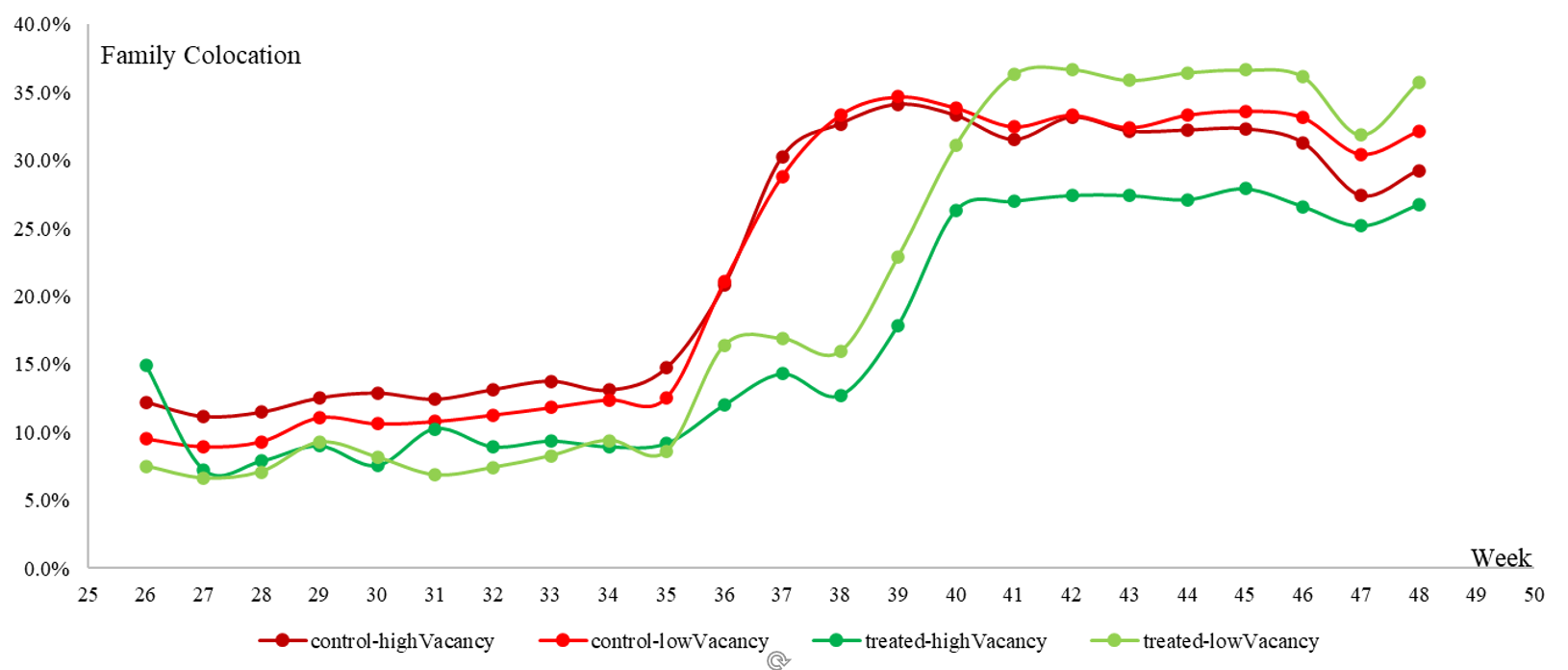

Supplement: S3 Fig — (TIF) [file pone.0273307.s012.tif]

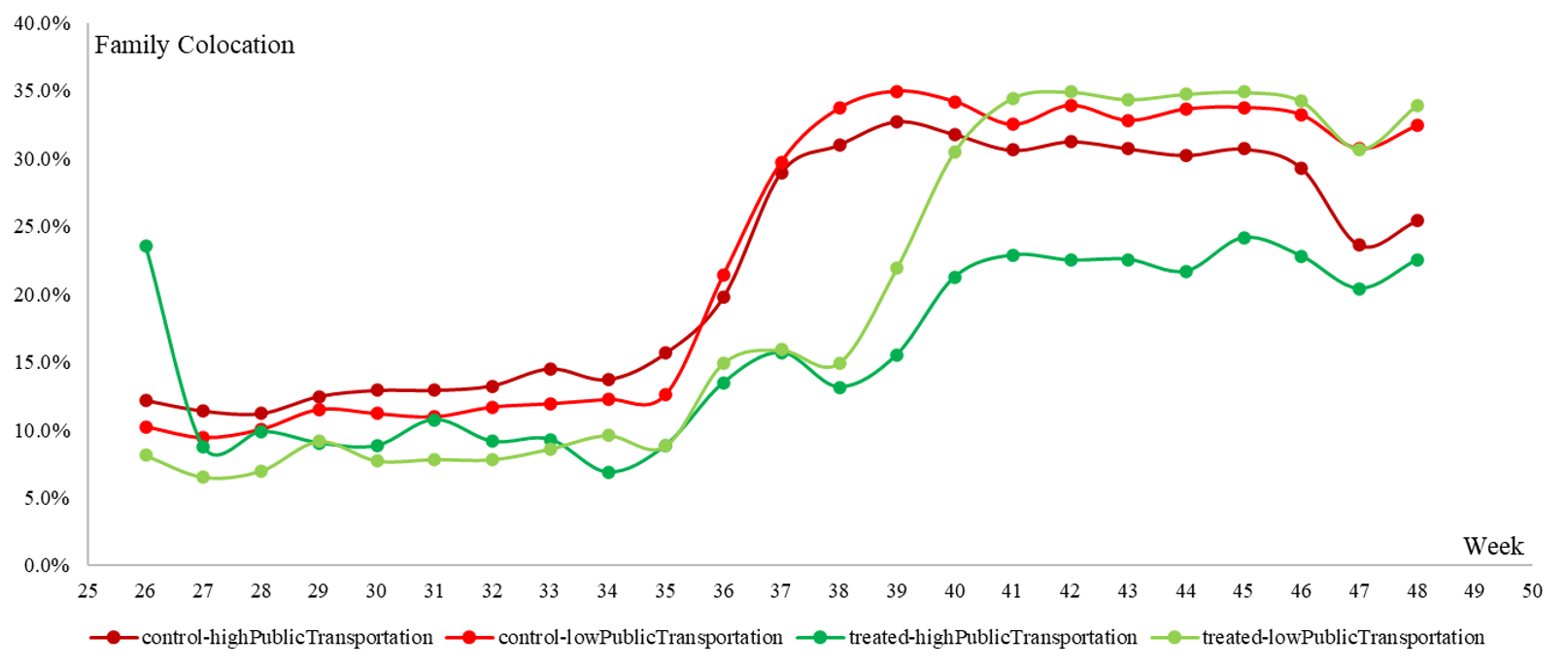

Supplement: S4 Fig — (TIF) [file pone.0273307.s013.tif]
